# Three-Dimensional Integrated Fan-Out Wafer-Level Package Micro-Bump Electromigration Study

**DOI:** 10.3390/mi14061255

**Published:** 2023-06-15

**Authors:** Wenchao Tian, Ran Gao, Lin Gu, Haoyue Ji, Liming Zhou

**Affiliations:** 1 Hangzhou Institute of Technology, Xidian University, Hangzhou 311231, China; gaoran0611@163.com; 2 School of Electro-Mechnical Engineering, Xidian University, Xi’an 710071, China; cameljihy@126.com; 3 Zhongkexin Integrated Circuit Co., Wuxi 214035, China; gulin110@163.com; 4 Yangzhou Yangjie Electronic Technology Co., Ltd., Yangzhou 225008, China; liming.zhou@21yangjie.com

**Keywords:** advanced packaging, electromigration, reliability, failure life

## Abstract

To meet the demands for miniaturization and multi-functional and high-performance electronics applications, the semiconductor industry has shifted its packaging approach to multi-chip vertical stacking. Among the advanced packaging technologies for high-density interconnects, the most persistent factor affecting their reliability is the electromigration (EM) problem on the micro-bump. The operating temperature and the operating current density are the main factors affecting the EM phenomenon. Therefore, when a micro-bump structure is in the electrothermal environment, the EM failure mechanism of the high-density integrated packaging structure must be studied. To investigate the relationship between loading conditions and EM failure time in micro-bump structures, this study established an equivalent model of the vertical stacking structure of fan-out wafer-level packages. Then, the electrothermal interaction theory was used to carry out numerical simulations in an electrothermal environment. Finally, the MTTF equation was invoked, with Sn63Pb37 as the bump material, and the relationship between the operating environment and EM lifetime was investigated. The results showed that the current aggregation was the location where the bump structure was most susceptible to EM failure. The accelerating effect of the temperature on the EM failure time was more obvious at a current density of 3.5 A/cm^2^, which was 27.51% shorter than 4.5 A/cm^2^ at the same temperature difference. When the current density exceeded 4.5 A/cm^2^, the change in the failure time was not obvious, and the maximum critical value of the micro-bump failure was 4 A/cm^2^~4.5 A/cm^2^.

## 1. Introduction

In recent years, in order to meet the needs of miniaturization and multi-functional and high-performance applications for electronic products, the requirements for microelectronic packaging have increased. When improving the performance of microelectronic systems, it is necessary to reduce the size and cost, which brings great challenges to traditional wafer-level packaging. As the traditional fan-in package is no longer able to complete the multilayer rewiring and bump array arrangement in its chip area, fan-out wafer-level packaging (FOWLP) has emerged. By increasing the area of a single package through wafer reconfiguration, the fan-out package breaks the limitation of the number of I/O terminals. Then, the advanced manufacturing process of wafer-level packaging is applied to complete the multilayer rewiring and bump preparation, in addition to cutting and separating, to obtain a package that can interconnect with external electrical properties [1,2,3].

Fan-out wafer-level packaging (FOWLP), an advanced packaging technology developed in recent years, eliminates the lap wire and flip chip bumps in traditional packaging structures and replaces the traditional PCB substrate with the redistribution layer (RDL). It has the advantages of high integration, high bandwidth, and short interconnection and is gradually developing into the mainstream technology of packaging [4,5,6,7]. On the other hand, FOWLP faces many critical issues to be solved in terms of reliability, such as multilayer-interconnect migration, large residual stresses, bump voids, bonding failures, and other failure behaviors. In advanced packaging technologies for high-density interconnects, EM problems on micro-bumps are the most persistent cause of reliability. Micro-bumps function as electrical and mechanical connections between FOWLP components, and their integrity and reliability are critical factors for the stable operation of electrical components [8]. While achieving high-density interconnections, current density aggregation and increased Joule heating have led to current congestion and rising temperature problems on micro-bumps [9,10,11,12]. Therefore, the operating temperature and operating current density are the main factors affecting the EM phenomenon on micro-bumps. Electromigration is a phenomenon in which atoms migrate rapidly with electrons under the action of high-density currents, creating voids or bumps and leading to component failure within the micro-bumps [13,14]. With the development and application of three-dimensional integration technology, the electromigration phenomenon in micro-bumps has attracted much attention [15,16]. Compared to other packaging technologies, FOWLP has a smaller package size and, therefore, is in a denser arrangement of micro-bumps. In the high-density interconnections of FOWLP, the electromigration failure phenomenon is highlighted due to the interaction of heat and electricity [17,18,19,20,21]. At present, the analysis of EM failure mechanisms for micro-bump structures is mostly based on experimental phenomena. In the literature [22], an interconnection model was developed for GaInSn/Cu materials. By applying different current densities to the model, the EM phenomenon under the current flux was obtained, which verified the migration of atoms from the cathode to the anode through the GaInSn path. In addition, the room temperature EM critical current density of Cu/GaInSn/Cu was two orders of magnitude lower compared to other EM systems. In multi-physical field conditions, the literature [23] studied the EM behavior caused by current crowding in Cu/Sn3.0Ag0.5Cu/Cu ball grid array (BGA) solder joints. By loading the current density and temperature conditions, it predicted the cavity formation location and critical value. The literature combines experimental phenomena to analyze the critical value of cavity formation from the perspective of atomic concentration. It does not analyze the relationship between loading conditions and lifetime in depth.

In this study, in order to obtain the relationship between the loading conditions and lifetime, the failure mechanism of the micro-bump EM phenomenon was investigated in a multi-physical field environment. Firstly, developing an equivalent model for the micro-bump structure of a high-density integrated package and its failure behavior was numerically simulated in an electrical-thermal environment. Then, the data were substituted into the MTTF equation, with Sn63Pb37 as the bump materials to predict the law, which is about the EM life of electronic products in relation to the loading conditions. Due to the adoption of the equivalent model of the advanced package form and using a high-density micro-bump interconnection structure as the research object, the research content will provide an important reference for extending the lifetime and improving the reliability of advanced packaging components.

## 2. Theoretical Analysis and Model Design

### 2.1. Electrothermal Interaction Theory

Under single environmental stresses, such as temperature cycling, high temperatures, EM, and mechanical stresses, the study of failure behavior and the development of life models of micro-bumps are gradually being advanced and improved [24]. However, under complex practical working conditions, it is difficult to singularize the effects of various environmental stresses by primary and secondary effects. From the perspective of close to the actual working conditions, it is necessary to explore the reliability of the micro-bump structure under multi-physical fields. When loading electronic products with both current and temperature conditions, the electromagnetic heat generated by the current will increase the temperature. Because of this, electrical–thermal conditions can have an impact on the heat transfer environment. In studying the relationship between loading conditions and lifetime, a theoretical model of the electrical–thermal interaction is required.

When an operating current is applied, the current density *j* follows the current conservation equation:

(1) $$j=(\sigma +\varepsilon_{0}\varepsilon_{r}\frac{\partial }{\partial t})E+j_{e},$$

(2) E=∇V,

(3) $$\sigma =\sigma_{0}\left(1+\partial \left(T-T_{0}\right)\right),$$

where σ is the material resistivity, $j_{e}$ is the external current density, $\varepsilon_{0}$ is the vacuum dielectric constant, $\varepsilon_{r}$ is the relative dielectric constant, ∇V is the potential gradient, $\sigma_{0}$ is the resistivity at the temperature $T_{0}$, and ∂ is the material temperature coefficient.

During the numerical simulation, the temperature field follows the heat transfer equation:

(4) $$\rho C_{p}\vec{u}\cdot \nabla T+\nabla \vec{q}=Q+Q_{ted},$$

(5) $$\vec{q}=-k\nabla T,$$

where ρ is the material density, *C_p_* is the constant pressure heat capacity, *q* is the heat flux density, *Q* is the heat, *Q_ted_* is the thermo-elastic damping, and *k* is the thermal conductivity.

In the electrothermal environment, current flow generates Joule heat, which interacts with the temperature field. The current and temperature field produce electromagnetic–thermal effects, following the electromagnetic heat equation for a multi-physics field:

(6) $$\rho C_{p}\vec{u}\cdot \nabla T=\nabla \cdot (k\nabla T)+Q_{e},$$

(7) $$Q_{e}=\vec{J}\cdot \vec{E},$$

where ρ is the material density, *C_p_* is the constant-pressure heat capacity, *k* is the thermal conductivity, and *Q_e_* is the heat generated by the electric field.

### 2.2. Numerical Simulation Model Construction

**Figure 1:**
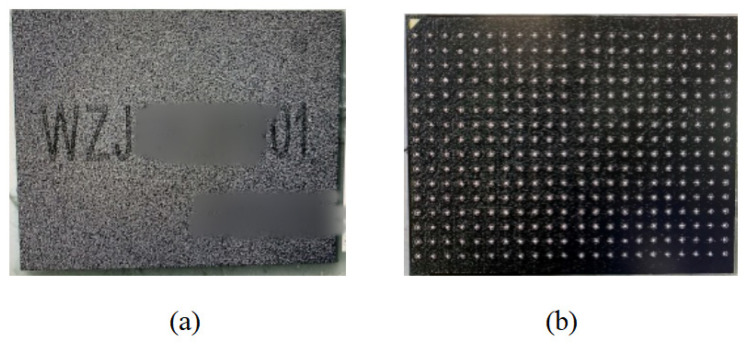
DDR3 micro-assembly physical diagram: (**a**) top component; (**b**) bottom component.

FOWLP maximizes the interconnect density and enables the seamless connection of semiconductor devices through two-to-five-micron spaced lines. At the same time, achieving high-bandwidth data transmission and removing the substrate results in significant cost savings. In addition, in the field of advanced packaging, high-density integrated packages with built-in vertical stacks are more prone to micro-bump EM failure problems. For this reason, a typical top-and-bottom vertically stacked micro-assembly structure was chosen as the experimental model. The actual model comes from DDR3 micro-assemblies produced by the 58th Research Institute of China Electronics Technology Group Corporation. DDR3 has a higher external data transfer rate and a more advanced topology architecture. On the other hand, it further reduces power consumption while ensuring performance. It used two 2 Gb DDR3 bare cores inside the overall micro-assembly model. The package structure was integrated with TSV technology on both sides, with vertical stacking of the top and bottom of the chip. Figure 1 shows the physical diagram of the micro-assembly.

**Figure 2:**
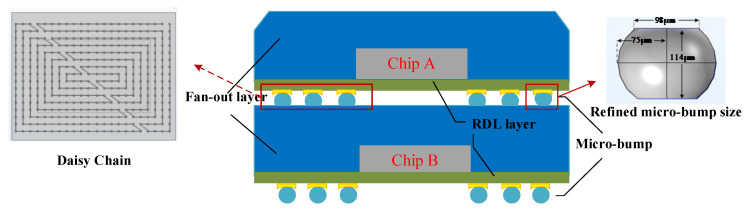
Schematic diagram of DDR simplified model.

The two DDR3s died in the micro-assembly, were pin-processed using fan-out technology and wired based on the redistribution layer (RDL) process. The model had a theoretical total capacity of 4 Gb and a bit width of 32 bits. Without affecting the simulation results, simplifying the model structure can reduce the workload and improve the calculation accuracy. Since the main object of this numerical simulation was the micro-bump structure, the TSV structure on both sides was simplified. The specific model was constructed as shown in Figure 2.

For the overall model, the micro-bump was the focus and difficulty, which needed to be refined. The size of the micro-bump changed in a thermo-electric coupled environment. Therefore, adjustments were made to the modeled size of the bump, as shown in Figure 2. In order to obtain the current density distribution that meets the engineering reality, a daisy chain structure was added to the micro-bump part. The daisy chain structure is made of Cu material. For forming a pathway between the micro-bumps, a wrap-around type complete connection was constructed between the entire micro-bump structure. Between the micro-bumps, the conductive properties of the material and the daisy chain structure design can realize the effective current flow. The simplified micro-assembly structure consisted of two chips (Si) and a fan-out package structure (polyimide, PI) stacked vertically, two RDL layers equivalent to a thin plate structure [25,26], and two 16 × 22 micro-bump structures (Sn63Pb37) on the top and bottom. The specific dimensions are shown in Table 1.

**Figure 3:**
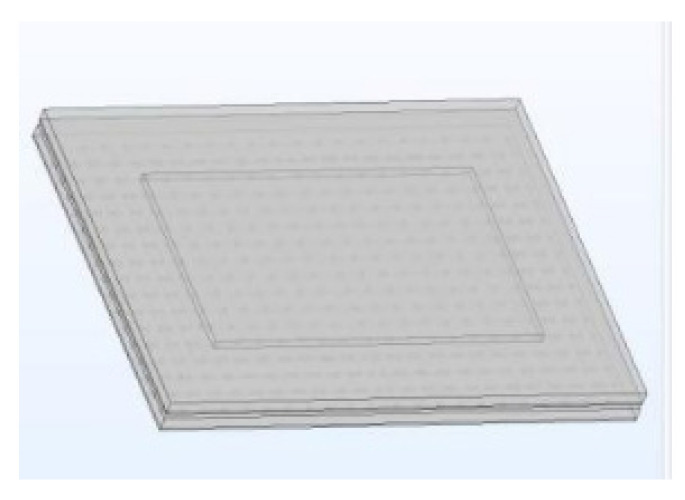
Micro-component Equivalent Model.

According to the dimensional parameters and the refined structural design of the key parts, the equivalent numerical simulation model was constructed. The specific model is shown in Figure 3.

## 3. Electrothermal Environmental Loading Conditions

### 3.1. Material Parameters

The chip layer, fan-out layer, and micro-bump were composed of a single material, and the material parameters used are shown in Table 2. The RDL structure consists of multilayer wiring, whose components mainly include PI and Cu. When assigning materials, the error of using a single material property is large. In order to ensure the accuracy of the electrical and thermal conductivity properties of the RDL layer, the material properties were obtained using the volume percentage method [27,28]. The volume percentage method is the most common method to determine the properties of equivalent materials, which can be obtained with isotropic and homogeneous properties. Based on the composition of the DDR micro-assembly rewiring layer, the PI was calculated to account for 57% and Cu for 43%. According to this ratio, the RDL layer material properties were obtained. The material parameters used in the model are shown in Table 2.

Since Sn63Pb37 is the key material to be studied in the electrothermal environment, its thermal conductivity properties are particularly important. The thermal conductivity of Sn63Pb37 as a bump material at different temperatures is labeled in Table 3.

### 3.2. Mesh Division and Boundary Conditions

**Figure 4:**
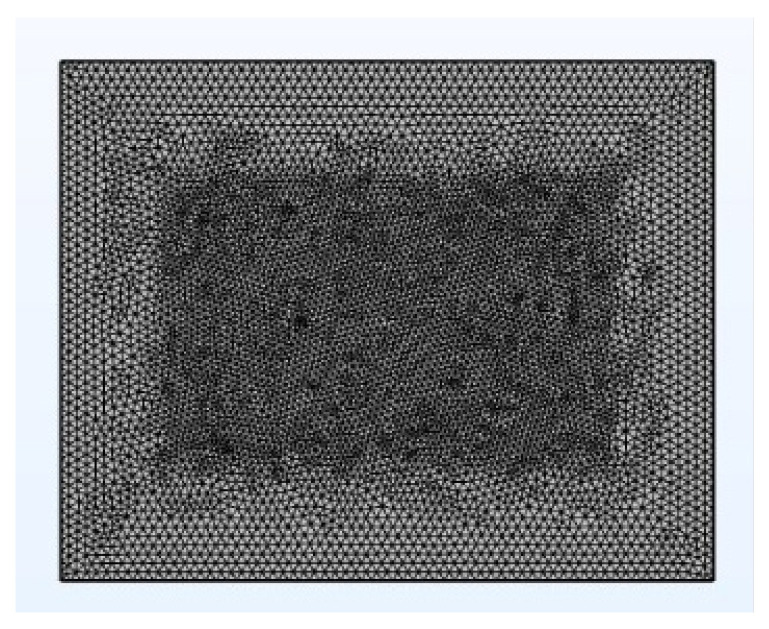
Overall meshing.

Referring to the experimental case of DDR micro-assembly, the overall temperature constraint (temperature = 398.15 K (corresponding to 125 °C)) and thermal convection constraint (natural convection heat transfer coefficient of 20 W/(m^2^·°C)) were imposed. A current density of 4 A/cm^2^ was applied at the chip end, which flowed into the micro-bump end through the RDL layer. In addition to this, a ground constraint was applied below the micro-bump. The overall meshing is shown in Figure 4, with 950,778 mesh vertices and 4,649,295 cells. The average quality of the mesh is around 0.7. The closer the mesh quality is to 1, the more accurate the calculation result.

**Figure 5:**
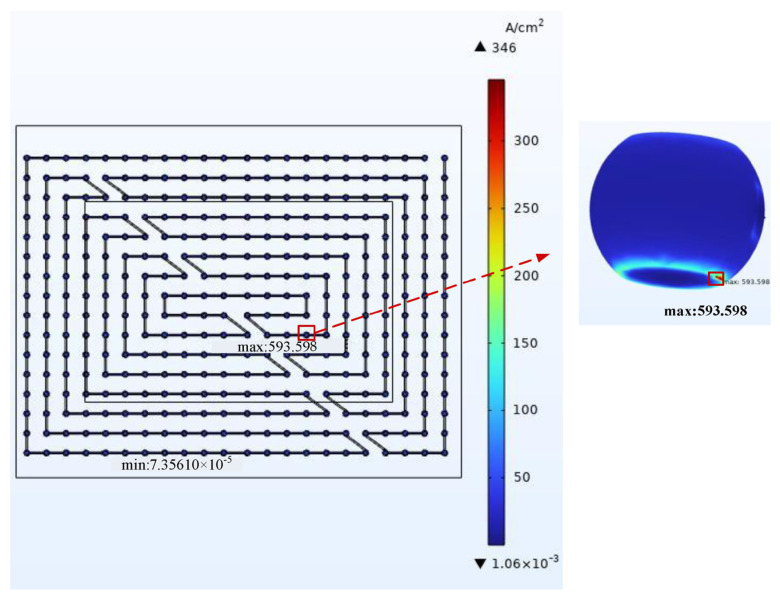
Current density at current aggregation: 593.60 A/cm^2^.

**Figure 6:**
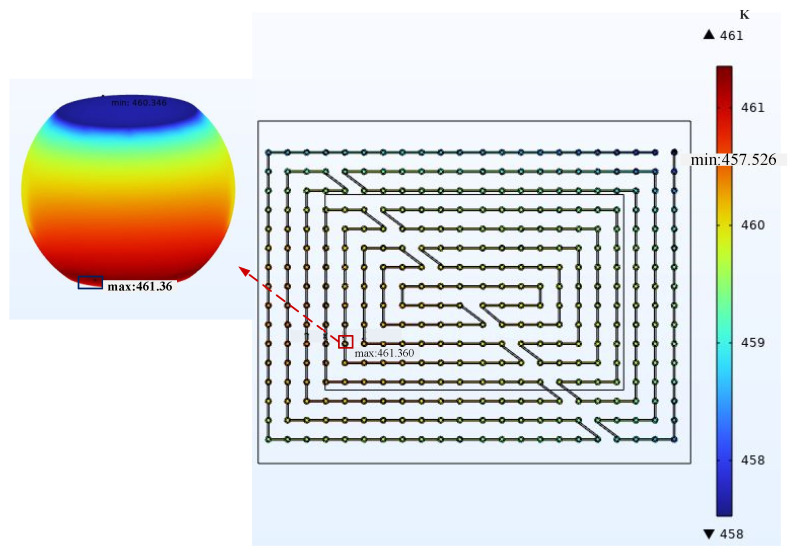
Temperature at hot spot: 461.36 K.

After completing the meshing, the simulation calculation was carried out on the model. The overall current density distribution and temperature distribution are shown in Figure 5 and Figure 6. From the result cloud diagram, it can be seen that the local current dense point location appeared in the middle area of the chip at the RDL layer and the bump connection, which is consistent with the actual current flow direction. The current density was 593.60 A/cm^2^. The highest local temperature point location appeared at the edge of the chip, and the temperature at the hot spot was 461.36 K.

**Figure 7:**
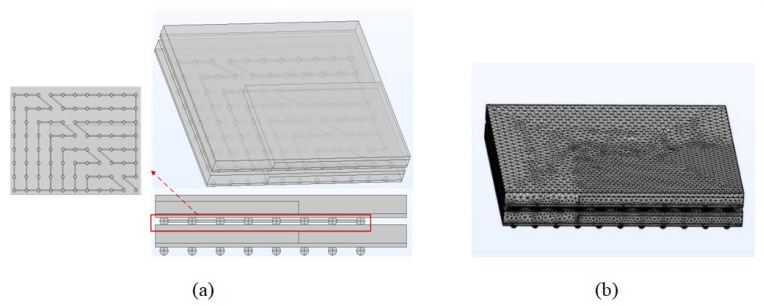
The 1/4 micro-component model: (**a**) three-dimensional diagram; (**b**) divided grid diagram.

The overall DDR model simulation was roughly calculated for the current density and temperature distribution. Since the heat generation at the chip was not negligible, the location distribution of current gathering points and hot spots was in accordance with the electric–thermal interaction principle. In order to improve the mesh quality, more accurate calculation results of key bumps can be obtained and the workload streamlined, while the 1/4 micro-component model can be used for subsequent work. The daisy chain structure was reset to the wraparound mutual circulation form. Symmetry constraints were added to the sub-model in order to make the results fit the DDR micro-component operating context. After fine mesh division, 325,534 mesh vertices, 1,629,738 cells, and an average mesh quality of 0.8 were available. The 1/4 micro-component model construction and the mesh division are shown in Figure 7.

This study loaded the model with an electrical-thermal environment using a numerical simulation. The simulation software used COMSOL, which could work on the principle of electrical–thermal interaction and imposed boundary conditions in conjunction with the actual operating conditions. The operating temperature of the micro-assembly was −40 °C~85 °C, and the operating voltage was in the range of −0.4 V~1.975 V. Therefore, 55 °C (328.15 K) and 125 °C (398.15 K) were selected as the normal operating temperature and accelerated experimental temperature, respectively. In the selection of current density, there were differences between experimental and numerical simulations. Based on the micro-assembly actual measurement dataand combined with the normal operating voltage range, 3.5 A/cm^2^~5 A/cm^2^ interval 0.5 was selected as the current density [22,29]. In addition to this, when the current critical value is reached, the change in MTTF will be small as the current density changes. The predicted normal operating current maximum was within this range. To simulate the natural heat dissipation environment, thermal convection constraints (natural convection heat transfer coefficient of 20 W/(m^2^·°C)) were still added. The specific loading conditions are shown in Table 4.

At the selected electrothermal environment, the micro-bump simulation results at both the operating and accelerating temperatures can be observed. Moreover, these experimental phenomena varied greatly with the current density at the same interval.

### 3.3. Theoretical Model for Electromigration Lifetime Prediction

The EM failure mechanism of micro-bump structures is the mass migration of atoms under the action of current stress. In order to obtain more accurate EM failure times, a thermo-electric reliability model needs to be constructed for Sn63Pb37. The conventional MTTF theory was proposed by Black in 1969, which analyzes the EM lifetime prediction [30].

(8) $$MTTF=A\frac{1}{(j{)}^{n}}\exp\frac{E_{a}}{kT},$$

where *MTTF* is related to the current density *j* and temperature *T* through three parameters, *Ea* is the activation energy of atomic diffusion in EM, *A* is the coefficient value, and *n* is the current density power factor. In general, both *A* and *n* are empirical valuations under practical applications.

The current flow will have Joule heating, and the entropy generated by this process is waste heat. The accumulation of entropy is carried out through the EM of atoms. Therefore, to revisit Black’s equation, the equation is considered in terms of entropy production in irreversible processes. Consider the link between entropy production and microstructural changes under mechanical damage. The theory of Onsager’s entropy yield in irreversible processes is [31]:

(9) $$\frac{TdS}{Vdt}=JX_{e},$$

where *T* is the temperature, $\frac{dS}{dt}$ is the entropy yield, *V* is the volume of the specimen, and *J* and *Xe* are the electron wind flux and driving force, respectively.

In EM, we consider the *MTTF* as the accumulation time of the threshold entropy (*S_threshold_*). According to Equation (9), the total entropy production up to the failure is derived as follows:

(10) $$t^{failure}=MTTF=\frac{TS_{threshold}}{VJ_{e}X_{e}},$$

where *J*_e_ is the electron wind flux, *Xe* is the driving force, and *D* is the atomic diffusivity. The *MTTF* is derived as follows:

(11) $$J_{e}=\frac{cD}{kT}Z^{*}e\;\rho \;j,$$

(12) $$X_{e}=Z^{*}e\;\rho \;j,$$

(13) $$D=D_{0}\exp(-\frac{Ea}{kT}),$$

(14) $$MTTF\approx t^{failure}=\frac{TS}{VJ_{e}X_{e}}=A\prime \frac{1}{j^{2}}\frac{1}{D}=Aj^{-2}\exp(\frac{Ea}{kT}).$$

From the derivation process above, the empirical value of Black’s equation can be defined as *n* = 2. One of the most widely used bump materials is Sn63Pb37. Finding the general A value of this material can quickly and accurately predict the EM lifetime of electronic products. The EM parameters of Sn63Pb37 are shown in Table 5.

The value of coefficient A of the MTTF equation has a significant impact on the electrical migration failure time. In the electric-thermal environment, concluding the first failure time of electronic products under different working conditions and the A value are obtained by substituting *n* = 2. According to the literature [34], the Weibull distribution of the MTTF data was obtained. By reading the article’s logic, it was found that there were data that clearly did not match the engineering reality. Following the article [35], the data were optimized within a reasonable range, and the experimental data of Sn63Pb37 were obtained. Next, the MTTF data from the Weibull distribution were quoted, and the A-value was calculated according to Equation (14).

It was calculated that the five sets of A-value data for this material in the literature [20] were calculated within the same order of magnitude. When the data error is not significant, the average value can be used for theoretical guidance. The average value is of the theoretical guidance. As a consequence, the A-value in the MTTF equation for Sn63Pb37, as a bump material, is 3.64 × 10^−3^. Substituting the A-value into Equation (14) yields Black’s equation for Sn63Pb37 as:

(15) $$MTTF=3.64\times 10^{-3}j^{-2}\exp(\frac{Ea}{kT}).$$

Equation (15) is the established reliability model for life calculation, which provides the theoretical basis for finding the average failure time of the micro-bump structure. Finally, the data are used to analyze the current density and temperature distribution in relation to the lifetime of the micro-bump.

## 4. Discussion

**Figure 8:**
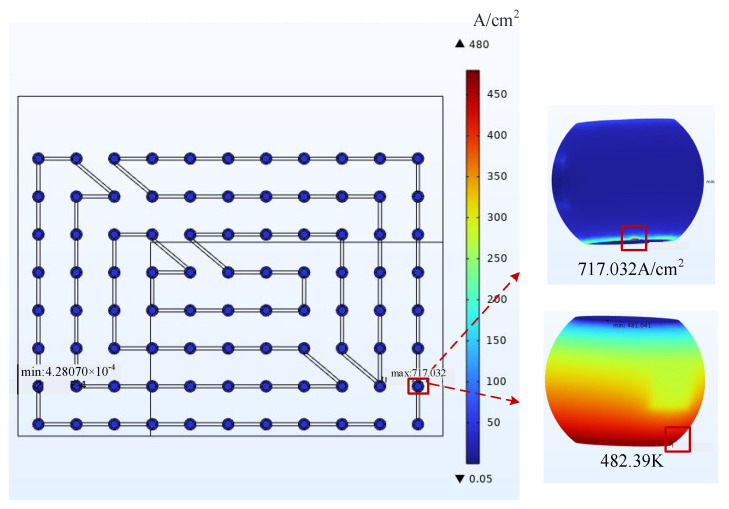
Cloud plot of the results at the current aggregation.

**Figure 9:**
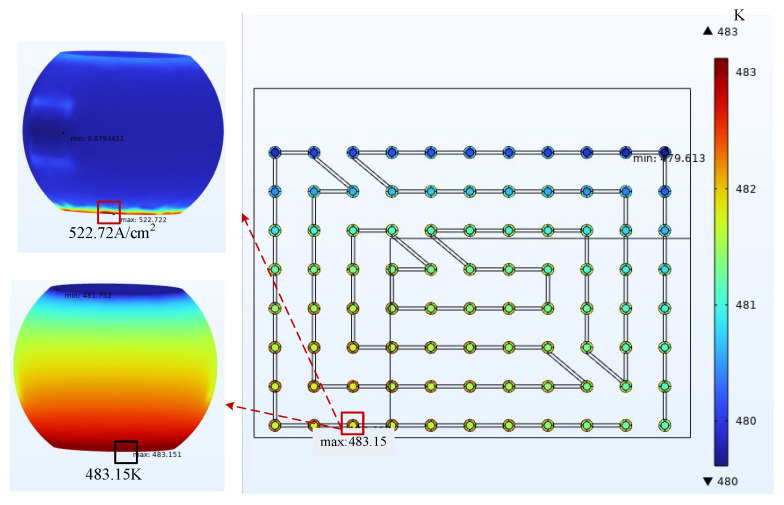
Resulting cloud at hotspot.

By observing the simulation results, the dangerous bumps that are most prone to EM failure were identified. The current density distribution and temperature distribution of current aggregation points and hot spots were counted, and the MTTF was calculated using Equation (15). After the mesh quality was improved, the simulation data of the overall model and the sub-model under the same working conditions were compared. The ambient temperature was 398.15 K, the natural convection heat transfer coefficient was 20 W/(m^2^·°C), and the current density applied at the chip end was 4 A/cm^2^. Figure 8 and Figure 9 show the resultant clouds at the current aggregation points and hot spots.

**Figure 10:**
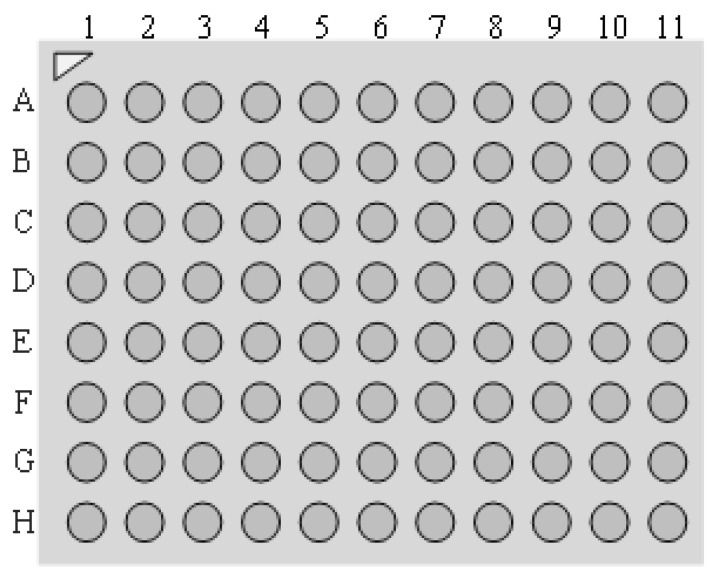
Sub-model bump coordinates.

By observing the cloud diagram, it can be directly seen that in the simulation calculation of the 1/4 model, the maximum current density was 717.03 A/cm^2^, and the hot spot temperature was 483.15 K compared to 593.60 A/cm^2^ and 461.36 K of the overall model in the same environment, respectively. The calculation results were more accurate after the grid quality was improved. In order to specifically describe the location of the hazard bumps, the coordinates of the bumps were set for the sub-model as in Figure 10. The highest local temperature points could be obtained at points H2, H3, and the local current gathering point at point G11 after the electrical–thermal simulation.

In the sub-model, the location of the current gathering point remained the same, however, the location of the highest local temperature point was shifted. According to the existing research, the failure phenomenon first occurs at the edges and corners when micro-convex structures are affected by the temperature [36,37]. The location of the highest local temperature point changed from the inside edge of the chip in the overall model to the outside edge. However, the maximum point of the temperature was always at the edge of the chip with a small offset. This indicates that when the simulation reached the steady state with increased computational accuracy, the effect of the temperature was more pronounced. The hot spot location did not exactly follow the current gathering point, and the effect of the temperature on EM failure came to the fore. The location of the current density aggregation point appeared to be consistent with the existing experimental phenomena [22].

Under different operating conditions, numerical simulations were carried out to obtain the current density distribution and temperature distribution. From the available studies in the literature, it can be concluded that dangerous solder joints exist at the maximum current and at the hot spots [38]. The locations of the dangerous bumps in the simulation results were found, and the values were counted. Then, the MTTF results were calculated using Equation (15). The simulation results and calculation results are shown in Table 6. For the same operating conditions, the first row shows the results at the current density, and the second row shows the results at the hot spots.

**Figure 11:**
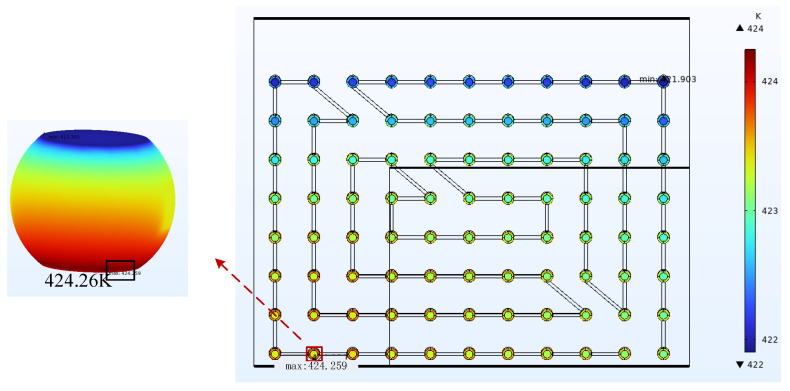
Hot spot temperature at 328.15 K and 3.5 A/cm^2^ is 424.26 K.

**Figure 12:**
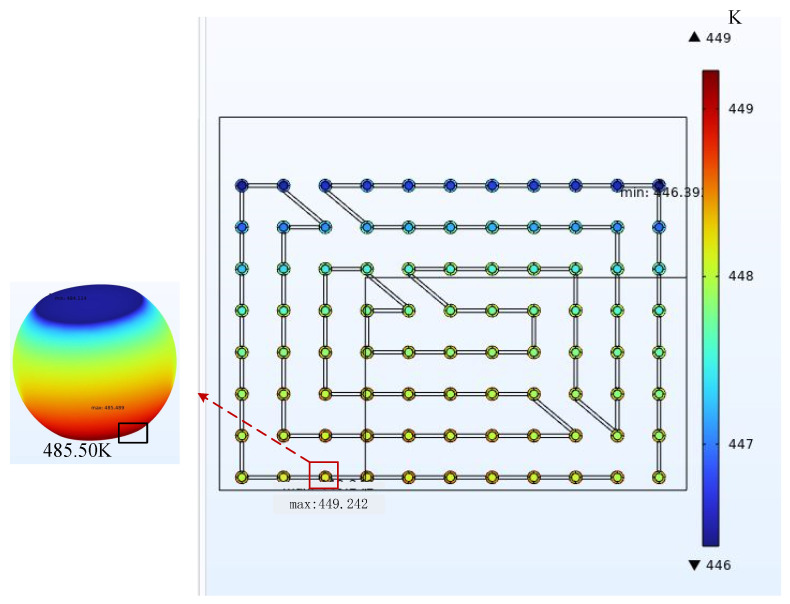
Hot spot temperature at 398.15 K and 3.5 A/cm^2^ is 485.50 K.

The data in the table above illustrate that under the same operating conditions, the calculated MTTF values at the current aggregation point in a steady state were smaller than those at the hot spot. Therefore, the influence of current density on the EM lifetime always dominates. The hot spot coordinates were shifted when the current densities were both 3.5 A/cm^2^ and the temperatures were 328.15 K and 398.15 K. The hot spot coordinates are H2 and H3, respectively. The results are shown in Figure 11 and Figure 12. This indicates that the hot spot locations were not fixed but moved within a certain range. When current density was applied to the chip to generate heat and add ambient temperature, the hot spot coordinates appeared at the location of the chip edge in the sub-model. When the current density dominates, the effect on the EM is greater as the temperature rises. Therefore, as the temperature changes, the hot spot location will move to a small area at the edge of the chip.

**Figure 13:**
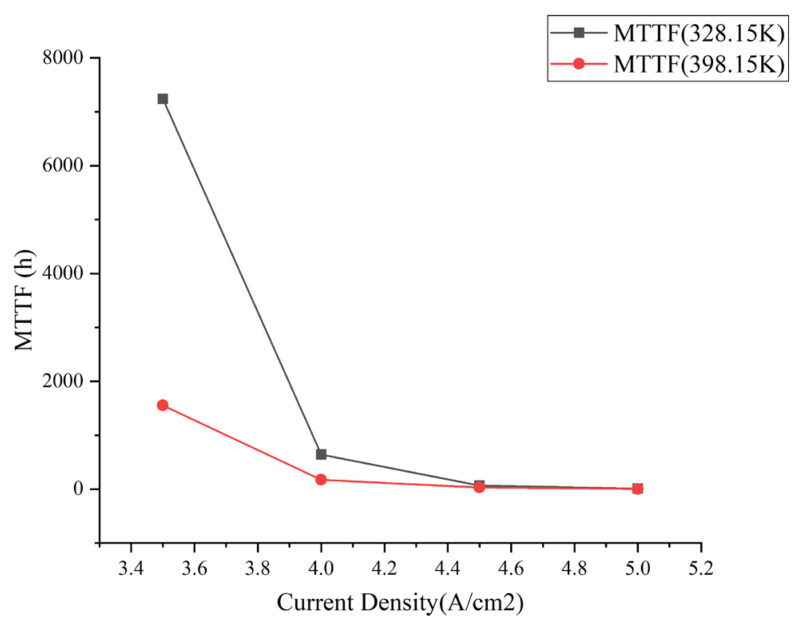
MTTF results comparison curve.

The normal operating temperatures of DDR components were −40 °C–85 °C (233.15 K–358.15 K). Therefore, the temperature for accelerated experimental conditions was 398.15 K. The MTTF was most obvious when the current density was 3.5 A/cm^2^, and the MTTF was shortened rapidly with the increase in temperature. Figure 13 shows the comparison of MTTF with the change in current under different temperatures.

By observing the trend in the comparison curves, it is found that the accelerating effect of the temperature on EM failure becomes less and less as the circuit density increases. When the temperature increased from 328.15 K to 398.15 K, and the current density was 3.5 A/cm^2^, the MTTF at the current aggregation changed from 7239.30 h to 1553.34 h. Comparing the MTTF at the two temperatures shows that the MTTF at 398.15 K was shortened by 21.46% at 328.15 K for a current density of 3.5 A/cm^2^. At the current density of 4.5 A/cm^2^, the MTTF at the current aggregation changed from 64.51 h to 31.59 h, and the MTTF at 398.15 K is shortened to 48.97% of that at 328.15 K. Therefore, at the same temperature difference, the MTTF decreased at a current density of 3.5 A/cm^2^ at a faster rate than at 4.5 A/cm^2^, with a 27.51% increase in the percentage decrease. Compared to the normal BGA package, the temperature variation and current density variation were more pronounced under the fan-out package [39]. From the comparison curves, it can be seen that the decreasing trend in MTTF changed the same when the temperature was different. The decreasing rate was the fastest at 3.5 A/cm^2^ to 4 A/cm^2^ and then gradually slowed down. It can be seen that the fundamental reason for the change in MTTF at different temperatures is that the increase in temperature intensifies the atomic migration rate, leading to faster EM failure.

When the temperature was 328.15 K, and the current density changed from 3.5 A/cm^2^ to 4 A/cm^2^, the MTTF at the current aggregation was changed from 7239.30 h to 643.84 h, which was shortened to 8.89% of the original. The data shows that the current density was the main influencing factor for the occurrence of EM failure. The experimental results are logically identical to those of the literature [34]. From the trend of Figure 13, it can be seen that as the current density increases to a certain value, its MTTF does not change significantly. At this time, the maximum threshold of the working current density has been reached. The maximum working current density of DDR micro-assembly is between 4 A/cm^2^–4.5 A/cm^2^. The MTTF decreased to about 97% of the original when the current density changed from 3.5 A/cm^2^ to 5 A/cm^2^, confirming that 5 A/cm^2^ has become an accelerated experimental condition. In addition, existing experimental studies have verified the correctness of the threshold current density [40].

## 5. Conclusions

In this study, the equivalence model was based on the DDR micro-assembly technology manual. Using the theory of electrothermal environment interaction, numerical simulations were performed in the electrothermal environment. According to the resultant plots, the current density distribution and temperature distribution of the hazardous bumps were obtained. The data were substituted into the MTTF equation for Sn63Pb37 to calculate the EM lifetime of the micro-bump structure. Finally, the relationship between the working environment and EM failure time was explored using statistical data.

The results showed that the most likely location for the EM failure of the bump structures was at the current aggregation. In the electrothermal environment, the current dominated the EM failure. The accelerating effect of the temperature on EM failure time was more obvious when the current density was 3.5 A/cm^2^, which was 27.51% shorter than 4.5 A/cm^2^ at the same temperature difference. In addition, when the current density exceeded 4.5 A/cm^2^, the change in failure time was not obvious, and the maximum critical value of the working current density of micro-bump structures was at 4 A/cm^2^–4.5 A/cm^2^.

Therefore, the study of the EM failure time of electronic products under different working conditions is extremely useful for exploring the relationship between the working environment and EM life. The conclusion helps to extend the EM life of the product represented by DDR3.

## Figures and Tables

**Table 1:** Dimensional parameters.

Components	Size (mm^3^)
Si	7.805 × 5.105 × 0.2
PI	11.3 × 8.95 × 0.3
RDL	11.3 × 8.95 × 0.063

**Table 2:** Material parameters.

Materials	Si	Cu	Polyimide	Sn63Pb37
Density (kg/m^3^)	2330	8940	1550	8425
Modulus of elasticity (Pa)	130 × 10^9^	255 × 10^9^	22 × 10^9^	34 × 10^9^
Coefficient of thermal expansion (ppm/K)	3.61	17	11.5	2.39
Poisson’s ratio	0.28	0.22	0.28	0.38
Thermal conductivity (W/m·K)	119	400	0.25	In Table 3
Resistivity (Ω·m)	2.52 × 10^−4^	1.72 × 10^−8^	1014.5	1.46 × 10^−4^
Specific heat capacity (J/(kg·°C))	712	385	1150	227.2
Relative dielectric constant	11.7	1	3.2	\

**Table 3:** Thermal conductivity of Sn63Pb37.

Temperature (K)	293	307.45	321.9	336.35	350.8	365.25	379.75	394.15	408.65	423.05
Thermal conductivity (W/mK)	52.02	51.89	51.76	51.63	51.5	51.37	51.24	51.11	50.98	50.85

**Table 4:** Electrothermal loading conditions.

Current Density (A/cm^3^)	3.5	3.5	4	4	4.5	4.5	5	5
**Temperature (K)**	328.15	398.15	328.15	398.15	328.15	398.15	328.15	398.15

**Table 5:** Electromigration parameters of 63Sn37Pb [32,33].

Electromigration Parameters	Numerical Value	Symbols	Unit
activation energy	1	*E_a_*	eV
Effective charge number	−33	Z*	/
Effective self-diffusion coefficient	3.14 × 10^−5^	*D0*	m^2^/s
Heat transfer	0.0094	Q^*^	eV
Atomic volume	2.48 × 10^−29^	*Ω*	m^3^
Initial resistivity	1.55 × 10^−7^	*R_0_*	Ω-m
Boltzmann’s constant	1.38 × 10^−23^	*K_B_*	J/K

**Table 6:** Experimental results and coefficient calculation for different working conditions.

Current Density (A/cm^2^)	Temperature (K)	Current Density Max (A/cm^2^)	Temperature Max (K)	MTTF (h)
3.5	328.15	627.37	423.74	7239.30
		394.31	424.26	17,721.10
3.5	398.15	627.4	448.97	1553.34
		457.38	449.24	2877.76
4	328.15	716.99	459.89	643.84
		450.64	460.62	1565.95
4	398.15	717.03	485.01	174.23
		522.72	485.49	320.18
4.5	328.15	806.62	500.88	64.51
		506.87	501.82	156.43
4.5	398.15	806.66	516.8	31.59
		507	517.93	76.16
5	328.15	896.24	546.56	7.54
		563.30	547.78	18.20
5	398.15	896.29	569.45	3.21
		563.34	570.84	7.73

## Data Availability

The data presented in this study are available upon request from the corresponding author.
